# Strategic Manufacturer Response to the Medicaid Rebate Cap Removal

**DOI:** 10.1001/jamahealthforum.2024.3624

**Published:** 2024-11-15

**Authors:** Joseph F. Levy, Mariana P. Socal, Jeromie M. Ballreich

**Affiliations:** 1 Department of Health Policy and Management, Johns Hopkins Bloomberg School of Public Health, Baltimore, Maryland

## Abstract

This economic evaluation illustrates a manufacturer’s estimated spending and revenues as it discontinued a branded product and introduced a generic replacement in response to a policy change.

## Introduction

State Medicaid receives pharmaceutical base and inflation rebates (eMethods in Supplement 1).^1,2^ Until recently, rebates were capped at the mean price manufacturers sell to wholesalers (average manufacturer price [AMP]). The American Rescue Plan removed the cap in January 2024 to discourage price increases above inflation, the primary reason a drug’s statutory rebate could exceed AMP.^3^ This change puts manufacturers at risk of having to pay Medicaid more than what manufacturers received for selling their products to wholesalers, resulting in net losses for each Medicaid sale.^4^

To elucidate choices manufacturers face due to this policy change, we examined the case of Flovent, a metered-dose fluticasone propionate inhaler produced by GlaxoSmithKline (GSK). In 2002, GSK launched an authorized generic (AG) of Flovent. On the day cap removal was enacted, GSK discontinued branded Flovent.

## Methods

We developed a model of Medicaid spending on Flovent for 2024 using historical data from 2005 quarter 2 (Flovent launch) through 2022 quarter 1 (before AG launch). This model used assumptions about statutory rebates, best price, and AMP that are not publicly disclosed (eMethods in Supplement 1). The model’s 4 scenarios were no cap removal (no AG launch) (A), cap removal with no response (B), policy’s intended response (C), and manufacturer’s actual response (D). In accordance with the Common Rule, this economic evaluation was exempt from ethics review and informed consent because it was not human participant research. We followed the CHEERS reporting guideline. Analysis was performed with Stata 16.1 (StataCorp LLC).

## Results

The Figure displays our estimated base and inflation rebates for Flovent from 2005 quarter 2 until 2022 quarter 1. Flovent hit the rebate cap around 2015 and exceeding it in all subsequent years. These disaggregated rebates and volume over time are the basis of our 2024 projections.

**Figure.**
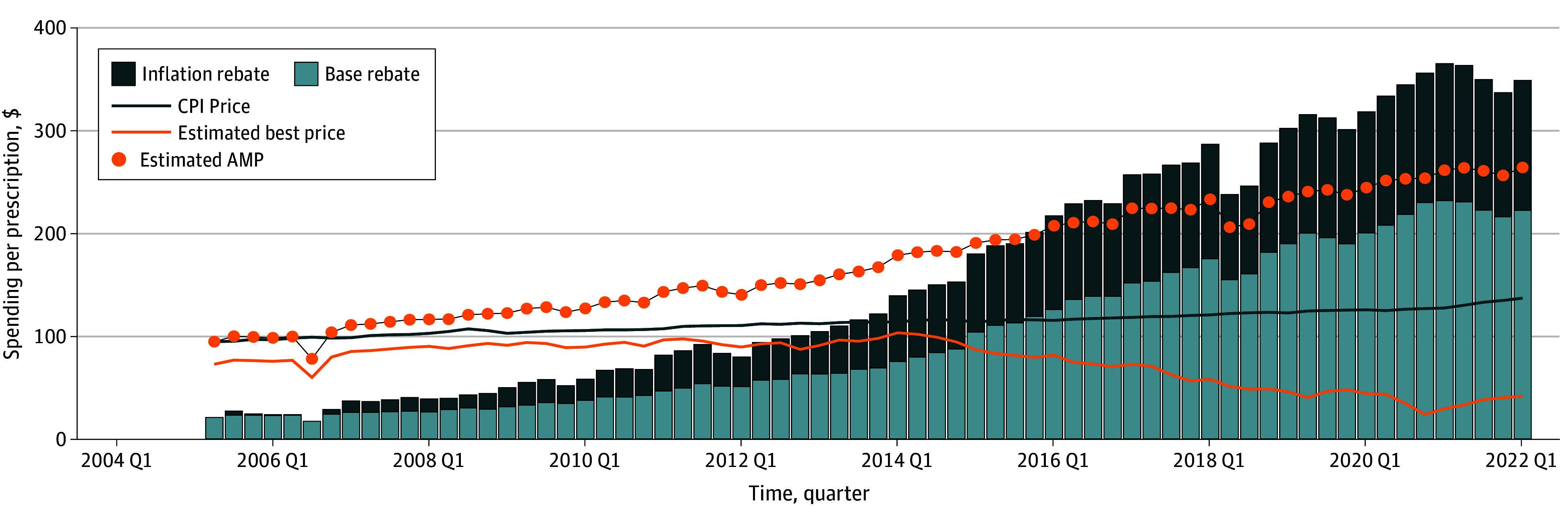
Quarterly Spending per Prescription in Medicaid AMP indicates average manufacturer price; CPI, Consumer Price Index.

The Table describes 2024 estimated spending. In scenario A, a projected 3 449 026 prescriptions would result in $1004.4 million in Medicaid gross spending, offset completely by $860.0 million in base rebates and $512.0 million in inflation rebates. Because scenario A continues the cap, total rebates would match gross spending, reducing net Medicaid spending to 0.

**Table.** Flovent Hydrofluoroalkane Spending in 2024 Under Different Scenarios^a^

Rebate cap scenario	Scenario strategic response	Retail spending per prescription, $^b^	Total spending, in millions, $	Base rebate, in millions, $^c^	Inflation rebate, in millions, $^d^	Actual rebate paid, in millions, $	Medicaid net spending to GSK, in millions, $^e^
No cap removal	A. No AG launch	291.21	1004.4	860.0	512.0	1005.0	0
Cap removal	B. No response	291.21	1004.4	860.0	512.0	1372.0	−367.6
Cap removal	C. Policy’s intended response: lower AMP to inflation level	150.70	519.8	434.9	0	434.9	84.9
Cap removal	D. Manufacturer’s actual response: launch Flovent AG; discontinue branded Flovent	183.90^f^	634.3	82.5	NA	82.5	551.8

Abbreviations: AG, authorized generic; AMP, average manufacturer price; GSK, GlaxoSmithKline; NA, not applicable.

^a^ N = 3 449 026 prescriptions. This linear projection of prescriptions was based on prescription fill data in Medicaid from 2005 to 2021 (prior to AG launch).

^b^ Based on gross sales per unit over time and projected to 2024.

^c^ Base rebate = maximum (estimated AMP – 23% or AMP – estimated best price).

^d^ Inflation rebate = Consumer Price Index–estimated AMP.

^e^ Negative numbers represent losses for GSK and gains for Medicaid only; no margin via acquisition cost to pharmacies was considered.

^f^ Spending was based on the National Average Drug Acquisition Cost in January 2024 (eMethods in Supplement 1).

In scenario B, Medicaid would receive $1372.0 million in rebates, exceeding the estimated gross spending. GSK would pay $367.6 million more to Medicaid than Medicaid paid to acquire the drug, making Medicaid net spending negative.

In scenario C, GSK would reduce the AMP to match the Consumer Price Index–adjusted price for 2024 quarter 1, eliminating the inflation rebate. Thus, Medicaid’s spending would decrease to $519.8 million. Rebates would total $434.9 million, for an $84.9 million net spending by Medicaid.

In scenario D, GSK would remove branded Flovent from the market, shifting use to the AG. Because generic rebates are calculated differently (13% of AMP), Medicaid net spending would increase to $551.8 million.

## Discussion

Results suggest that instead of continuing to sell Flovent to Medicaid for 0 revenue, GSK faced losing $367.6 million per year after the rebate cap removal. This amount was almost as high as Flovent’s total net US sales (approximately $394 million in 2022).^5^ Additionally, GSK did not lower the price to match inflation, which would have generated $84.9 million in spending. Instead, GSK discontinued branded Flovent and shifted use to the AG, which would have generated over $500 million in Medicaid spending or revenue for GSK.

A study limitation includes assuming consistent Flovent use, which was done to better compare financial implications across scenarios. However, evidence suggests that the AG of Flovent has lost market share because it has not received preferred formulary status by Medicaid and commercial plans. Nevertheless, the findings suggest that rebate cap removal had unintended consequences, preventing reductions in spending as projected.^6^ Additional policies, such as increasing statutory rebates for AGs beyond the 13% used for generics and tying inflation-adjusted measures to the branded price when an AG is launched, are warranted. Future analysis should ascertain whether cap removal affected launch prices and lifecycle pricing strategy.

## Notes

Supplement 1.
eMethods.

Supplement 2.
Data Sharing Statement
