# Supplementary material for: Strategic Manufacturer Response to the Medicaid Rebate Cap Removal
Source: JAMA Health Forum. 2024 Nov 15;5(11):e243624. doi: 10.1001/jamahealthforum.2024.3624 (PMC11568456; doi:10.1001/jamahealthforum.2024.3624)
Supplement: Supplement 1. — eMethods. [file jamahealthforum-e243624-s001.pdf]

Supplemental Online Content

Levy JF, Socal MP, Ballreich JM. Strategic manufacturer response to the Medicaid rebate cap removal. *JAMA Health Forum.* Published online November 15, 2024.
doi:10.1001/jamahealthforum.2024.3624

eMethods.

This supplemental material has been provided by the authors to give readers additional information about their work.

© 2024 Levy JF et al. *JAMA Health Forum*.

eMethods.

Rebates in Medicaid

Medicaid receives statutory rebates for branded pharmaceuticals. The rebate has two components: a base rebate and an inflation rebate. The base rebate effectively provides Medicaid with the same rebate the best-situated private plan receives, while the inflation rebate can increase the total rebate depending on how the price has evolved since launch relative to inflation. The total rebate amount was capped at 100% of the Average Manufacturers Price, which is the weighted unit price of purchases made by wholesalers from manufacturers (the first purchases in the supply chain).

The 2021 American Rescue Plan removed the cap, requiring manufacturers to pay a rebate greater than 100% of the AMP if the base rebate + inflation rebate exceeded the AMP for branded pharmaceuticals. The cap removal went into effect January 1, 2024.

Estimating Rebates for Flovent

We constructed a model of projected Medicaid spending on Flovent HFA in 2024. The model is built from historical data on utilization and assumptions about confidential numbers necessary to estimate rebates and, ultimately, spending. Data on prescription reimbursement and gross prices are derived from the Medicaid State Utilization Database 2005q1-2022q1 as this is the period Flovent HFA was sold prior to the introduction of Flovent HFA AG. We estimate rebates using two assumptions: first, that the Gross Sales that Medicaid pays at point of sale is reflective of the AMP, and second, that SSR Gross-to-Net percentage rebate times the estimated AMP approximates the Best Price for Flovent. Each rebate type, base and inflation are estimated quarterly and at the cost per average prescription level. Base rebates were estimated as the greater of {*Estimated AMP * 23.1%* or *Estimated AMP-Estimated Best Price}* per statute. Inflation rebates are estimated by taking the estimated AMP at launch and carrying forward at the rate of inflation. Taken together, these estimates allow us to understand when Flovent HFA likely hit the cap and identify how much would be owed with cap removal in 2024.

Estimating Medicaid Spending on Flovent in 2024

The spending model for 2024 examines four different scenarios based on the constructed historical trend, all parameters that were built from the trend prior to 2022q2 and estimated to progress linearly through 2024, except CPI, where the actual CPI trend was used. First, we modeled Medicaid spending absent cap removal, with no authorized generic launch or strategic response by GSK. This scenario (Scenario A) can be thought of as the spending situation for Medicaid on Flovent absent the cap removal. Next, modeling the removal of the cap, with no strategic response by the manufacturer (Scenario B) explains the situation presented to GSK with the cap removal estimating losses incurred to GSK (revenue to Medicaid).

Next, we present two scenarios of strategic response; the first (Scenario C), arguably the intent of the policy, is for GSK to lower the price of the drug (the AMP) to a level that does not exceed inflation since launch. Lowering to the exact amount the Flovent price relative to CPI is modeled negates any inflation rebate, and prevents a manufacturer from losing money selling the drug to Medicaid. Finally, (Scenario D) models spending on the authorized generic, with the removal of the branded product from market; which was the approach GSK ultimately adopted. Price for the Flovent AG was estimated as the NADAC

© 2024 Levy JF et al. *JAMA Health Forum.*

for the Flovent AG observed in January 2024, but utilization was assumed constant, and rebates off the AG (which statutorily defined as 13% of AMP, for generic drugs) were estimated to be 13% of NADAC.

Rebates are presented at the per-prescription level to help visualize the size of the rebate, removing the impact of utilization changes over time. The spending estimates are represented at the national annual level. Notably, utilization is assumed stable under all four models, i.e., there is no demand response to price or shifting to only AG Flovent being available.

© 2024 Levy JF et al. *JAMA Health Forum*.
