# Supplementary material for: Strategic Manufacturer Response to the Medicaid Rebate Cap Removal
Source: JAMA Health Forum. 2024 Nov 15;5(11):e243624. doi: 10.1001/jamahealthforum.2024.3624 (PMC11568456; doi:10.1001/jamahealthforum.2024.3624)
Supplement: Supplement 2. — Data Sharing Statement [file jamahealthforum-e243624-s002.pdf]

Data Sharing Statement

Levy. Strategic Manufacturer Response to the Medicaid Rebate Cap Removal. *JAMA Health Forum*. Published November 15, 2024. doi:10.1001/jamahealthforum.2024.3624

Data
Data available: No

Additional Information
Explanation for why data not available: Data can be shared by email request to jlevy@jhu.edu except for estimates derived from SSRHealth proprietary gross-to-net ratio quarterly over time, which we are not able to disclose due to the terms of our data use agreement.
